# Navigating Pediatric Cor Pulmonale: A Comprehensive Review of Diagnosis and Management

**DOI:** 10.7759/cureus.67782

**Published:** 2024-08-26

**Authors:** Yash Thakur, Revat J Meshram, Amar Taksande

**Affiliations:** 1 Pediatrics, Jawaharlal Nehru Medical College, Datta Meghe Institute of Higher Education and Research, Wardha, IND

**Keywords:** pediatric cor pulmonale, multidisciplinary care, diagnostic strategies, congenital heart defects, right ventricular dysfunction, pulmonary hypertension

## Abstract

Pediatric cor pulmonale, characterized by right ventricular dysfunction due to chronic pulmonary hypertension, presents significant diagnostic and management challenges. This comprehensive review delves into this complex condition's etiology, clinical presentation, diagnostic strategies, and management. Key etiological factors include congenital heart defects, chronic lung diseases, and pulmonary vascular disorders. Early diagnosis, facilitated by imaging, hemodynamic assessments, and laboratory investigations, is crucial for effective intervention. Pediatric cor pulmonale management encompasses pharmacological treatments, such as vasodilators, diuretics, and inotropic agents, and non-pharmacological interventions, including oxygen therapy, mechanical ventilation, and surgical options. Long-term follow-up is essential to monitor disease progression and adjust treatment strategies accordingly. Multidisciplinary care involving pediatric cardiologists, pulmonologists, and critical care specialists is paramount to address the multifaceted needs of these patients. The review highlights the importance of early recognition and comprehensive care, offering insights into current best practices and future research and clinical practice directions. Advances in understanding the pathophysiology of pediatric cor pulmonale and emerging therapies promise to improve patient outcomes, underscoring the need for continued collaboration and innovation in this field.

## Introduction and background

Pediatric cor pulmonale encompasses the spectrum of right ventricular dysfunction stemming from chronic pulmonary hypertension in pediatric patients. This condition arises as a consequence of various underlying causes, including congenital heart defects, chronic lung diseases such as cystic fibrosis or bronchopulmonary dysplasia, and pulmonary vascular disorders like pulmonary arterial hypertension [1]. The hallmark feature of pediatric cor pulmonale lies in the structural and functional alterations occurring within the pulmonary vasculature, leading to increased pulmonary vascular resistance. This chronic elevation in pulmonary pressure significantly burdens the right ventricle, resulting in hypertrophy as it struggles to overcome the increased afterload. Over time, this adaptation may progress to right ventricular failure, exacerbating symptoms and compromising overall cardiac function [2]. The importance of early recognition and management of pediatric cor pulmonale cannot be overstated. Timely identification of this condition allows for prompt initiation of therapeutic interventions aimed at mitigating pulmonary hypertension and preventing further progression of cardiac dysfunction [3]. Early intervention not only alleviates symptoms such as dyspnea, fatigue, and exercise intolerance but also plays a crucial role in improving long-term outcomes. Addressing the underlying pathological processes early in the disease course may halt or even reverse the progression of pulmonary vascular disease, preserving cardiac function and enhancing the quality of life for pediatric patients with cor pulmonale [4].

This comprehensive review is a definitive resource for healthcare professionals caring for pediatric patients with cor pulmonale. By systematically examining the etiology, clinical presentation, diagnostic modalities, therapeutic strategies, complications, and prognosis associated with this condition, this review aims to provide a thorough understanding of cor pulmonale in the pediatric population. Synthesizing current evidence-based practices and clinical insights, this review seeks to empower clinicians with the knowledge and tools necessary to optimize the diagnosis and management of pediatric cor pulmonale, ultimately improving patient outcomes and enhancing the quality of care delivered to this vulnerable patient population.

## Review

Etiology of pediatric cor pulmonale

Congenital Heart Defects

Congenital heart defects (CHDs) encompass structural abnormalities within the heart or major blood vessels that manifest at birth. These anomalies exhibit a spectrum of severity, ranging from simple issues such as "holes" between heart chambers to intricate malformations like the absence of chambers or valves [5-7]. CHDs can adversely affect the heart's capacity to pump blood efficiently, leading to aberrant blood flow, valve dysfunctions, or occluded blood flow within the heart or vessels [8]. While the precise etiology of CHD remains unclear in many cases, it can be associated with genetic factors, chromosomal abnormalities, and certain prenatal risk factors such as diabetes or specific medications [8]. The initial pathophysiologic event in the production of cor pulmonale is an elevation of pulmonary vascular resistance. As the resistance increases, the pulmonary arterial pressure rises, and the right ventricular work increases, leading to enlargement (e.g., thickening, dilation, or both). There exist various types of congenital heart defects, including atrial septal defects (ASD), coarctation of the aorta (CoA), tetralogy of Fallot, and ventricular septal defects (VSD), among others [7-9]. These defects can provoke cyanosis (a bluish skin hue), rapid breathing, poor feeding, dyspnea, and irregular heart rates or rhythms [8]. Diagnosis of CHDs can be established prenatally through fetal echocardiograms or postnatally through physical examinations, cardiac assessments, genetic evaluations, and imaging modalities such as echocardiograms, MRIs, and CT scans [8]. Management of CHDs may entail surgical interventions, catheter-based procedures, pharmacotherapy, and ongoing surveillance to effectively control the condition and enhance cardiac function [7].

Chronic Lung Diseases

Chronic lung diseases encompass a spectrum of respiratory disorders impacting children, particularly premature infants, leading to persistent breathing difficulties and lung complications. These conditions often entail lung damage, including scarring, inflammation, and tissue breakdown, which impede breathing in affected infants [10-12]. Among infants, bronchopulmonary dysplasia (BPD), also known as chronic lung disease (CLD), stands as a prevalent form of chronic lung disease, predominantly affecting premature newborns due to their vulnerable and easily susceptible lung tissue [10-12]. Indicative symptoms of chronic lung disease in infants encompass respiratory distress, rapid breathing, nostril flaring, grunting, and chest retractions, signaling the necessity for mechanical ventilation or oxygen supplementation beyond 36 weeks of gestation [11]. Diagnostic procedures typically involve chest X-rays to visualize lung alterations, blood tests to assess oxygen saturation levels, and echocardiography to exclude cardiac anomalies and confirm the presence of chronic lung disease [11]. Treatment modalities for chronic lung diseases in infants may encompass mechanical ventilation, continuous positive airway pressure (CPAP), pharmaceutical interventions, and oxygen therapy to facilitate breathing support and aid in lung recovery and development [12,13]. The etiology of chronic lung diseases in infants is multifaceted, with significant risk factors including prematurity, low birth weight, maternal infections, inflammation, and exposure to elevated oxygen concentrations [12,13]. Effective management of these conditions necessitates specialized care provided by healthcare professionals adept in monitoring and addressing the respiratory challenges affected infants encounter. This specialized care aims to ensure optimal growth and development while navigating the complexities of these chronic respiratory disorders [14].

Pulmonary Vascular Disorders

Pulmonary vascular disorders encompass a diverse array of conditions impacting the blood vessels within the lungs, with pulmonary hypertension (PH) emerging as a prevalent and grave complication [15]. These disorders can manifest from various origins, including congenital heart disease, pulmonary ailments, rheumatologic conditions, sleep apnea, liver disorders, prematurity, hematologic disorders, and exposure to specific medications [15]. The pathophysiology underlying pulmonary vascular disease involves aberrations in pulmonary blood flow, left atrial pressure, and/or pulmonary vascular resistance, culminating in pulmonary hypertension among children afflicted with congenital and acquired heart pathologies [16]. Accurate diagnosis of pulmonary vascular disorders necessitates a meticulous assessment to elucidate the underlying cause of pulmonary hypertension, excluding alternative etiologies, and delineate a risk profile, with cardiac catheterization as a pivotal diagnostic modality [16]. Treatment approaches for pulmonary arterial hypertension associated with congenital heart disease (PAH-CHD) adhere to guideline recommendations. However, the evidence often derives from extrapolations of studies conducted on other etiologies of PAH due to the multifaceted nature of pulmonary hypertension in pediatric cardiac conditions [16]. Managing pulmonary vascular disorders in children can pose significant challenges, particularly when the etiology is multifactorial or unclassifiable, necessitating a holistic approach to address the complexities inherent in these conditions [16]. The therapeutic objectives in treating pulmonary hypertension center on alleviating symptoms, enhancing the quality of life, and addressing concomitant health exigencies such as cardiac and pulmonary failure and other associated conditions [15].

Neuromuscular Disorders Affecting Respiratory Function

Neuromuscular disorders profoundly affect respiratory function through various mechanisms, encompassing respiratory muscle weakness, hypotonia of bulbar muscles, anatomical anomalies like scoliosis, and diminished central respiratory drive [17-19]. These conditions predispose individuals to respiratory failure, characterized by compromised airway clearance, hypoventilation leading to hypoxia and hypercapnia, and ultimately, respiratory muscle weakness progressing to overt respiratory failure [17-19]. While the decline in respiratory function may manifest gradually, certain health complications, such as infections, can expedite the necessity for mechanical intervention, underscoring the importance of proactive management [17,20]. Individuals with neuromuscular diseases are susceptible to developing chronic respiratory failure due to degenerative muscle conditions, warranting vigilant monitoring and long-term therapeutic interventions [18]. Early manifestations of respiratory muscle weakness may include a soft breathy voice, slurred speech, weakened cough, morning headaches, and cognitive impairment, underscoring the significance of identifying these symptoms promptly for timely intervention [20]. Differential diagnosis and assessment of respiratory muscle weakness are critical, entailing comprehensive screening for underlying etiologies and appropriate follow-up measures to manage chronic respiratory muscle weakness effectively [18].

Other Contributing Factors

In addition to the previously discussed factors, pediatric cor pulmonale can be influenced by conditions such as obstructive sleep apnea (OSA) and pulmonary thromboses in acute respiratory distress syndrome (ARDS) [21,22]. In children with OSA, predisposing factors such as obesity, adenotonsillar hypertrophy, and craniofacial skeletal abnormalities contribute to the risk of cor pulmonale, with younger age and higher body mass index (BMI) serving as significant determinants [21]. Furthermore, the presence of hypoxemia and hypoventilation, commonly associated with obesity, further exacerbates the susceptibility to cor pulmonale in pediatric OSA patients [21]. Regarding pediatric ARDS, pulmonary thromboses are identified as potential contributors to cor pulmonale, with biomarkers indicating coagulopathy and endothelial injury playing pivotal roles in the pathophysiology of thrombotic phenomena in these cases [22]. Factors such as elevated plasminogen activator inhibitor-1, decreased protein C levels, and biomarkers of endothelial injury are associated with heightened mortality and morbidity in pediatric ARDS, underscoring the significance of coagulopathy in the development of pulmonary thromboses and subsequent cor pulmonale [22]. The multifactorial nature of pediatric cor pulmonale encompasses a combination of respiratory conditions like OSA and ARDS, alongside associated risk factors including obesity, hypoxemia, and coagulopathy, highlighting the intricate interplay of factors contributing to this condition in pediatric patients.

Clinical presentation

Signs and Symptoms of Pediatric Cor Pulmonale

**Figure 1:**
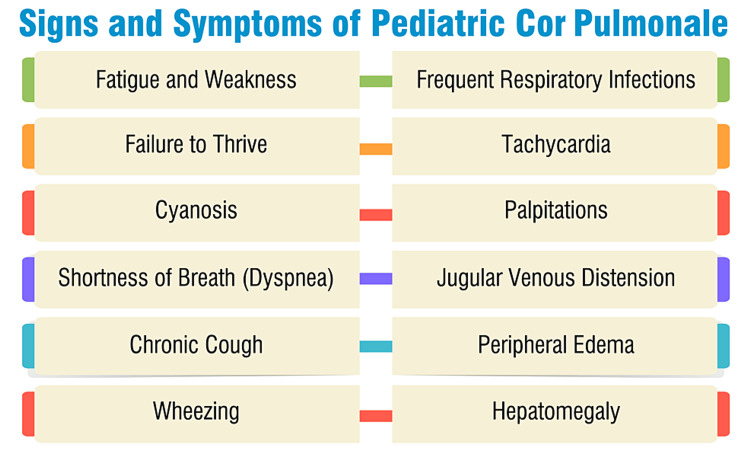
Signs and symptoms of pediatric cor pulmonale Image credits: Dr. Yash Thakur

Dyspnea, commonly known as shortness of breath, frequently arises due to heightened work of breathing attributed to alterations in lung elasticity or modified respiratory mechanics. Orthopnea and nocturnal dyspnea, although uncommon symptoms of right heart failure, manifest as a consequence of intensified work of breathing when in a supine position, resulting from compromised diaphragmatic excursion. Tussive or effort-related syncope, stemming from the right ventricle's incapacity to adequately supply blood to the left side of the heart, may also occur. Additional symptoms may encompass leg cramps, chest pain, a reversed blood pressure gradient in limbs, absent or faint femoral pulses, abnormal or amplified heart sounds, distended neck veins, peripheral edema in the legs and hands due to fluid retention, and hepatomegaly. These manifestations signify the impact of cor pulmonale on the right ventricle and the circulatory system, underscoring the significance of early recognition and appropriate management to ameliorate outcomes in pediatric patients [23]. The signs and symptoms of pediatric cor pulmonale are listed in Figure 1.

Variations in Presentation Based on Underlying Etiology

Variations in clinical presentation often stem from the diverse etiologies underlying a condition. Take cor pulmonale, for instance. Clinical manifestations may vary depending on the specific causative factors precipitating its development. Etiologies of cor pulmonale span from structural anomalies to genetic predispositions, thereby influencing the symptoms and signs displayed by affected individuals [24]. Similarly, the underlying etiological factors significantly shape the clinical presentation of acute and chronic pancreatitis. Variables such as alcohol abuse, smoking, gallstones, hypertriglyceridemia, and genetic mutations can exert profound effects on the manifestation of pancreatitis and its transition from an acute to a chronic state [25]. These divergent etiological influences contribute to disparities in the clinical features observed among patients with pancreatitis, underscoring the imperative of considering the underlying causes when evaluating and managing the condition.

Importance of Early Recognition and Differential Diagnosis

The significance of early recognition and differential diagnosis in cor pulmonale lies in the ability to promptly identify the condition and differentiate it from other potential causes presenting with similar symptoms. Early recognition is paramount as interventions initiated at an earlier stage may potentially reverse the pathological process or impede its progression, underscoring the critical nature of timely diagnosis and management in pulmonary vascular disease [26]. Differential diagnosis assumes a pivotal role in discerning between acute and chronic cor pulmonale, facilitating appropriate management and treatment selection predicated on the underlying pathophysiology and disease trajectory [27]. In cor pulmonale, the differential diagnosis entails consideration of conditions such as thromboembolic disease, primary pulmonary hypertension, blood disorders associated with augmented blood viscosity, congestive heart failure, high-output heart failure, infiltrative cardiomyopathies, primary pulmonic stenosis, right heart failure due to diverse causes, and other related conditions [27]. This comprehensive approach to differential diagnosis aids in the exclusion of alternative etiologies and directs attention toward specific management strategies tailored to cor pulmonale.

Diagnostic approach

History Taking and Physical Examination

History taking and physical examination hold fundamental importance in medical practice for diagnosing and managing patients effectively. History taking entails collecting information regarding the patient's symptoms, medical history, family history, social background, allergies, and current medications to understand the patient's health status [28] comprehensively. It serves as a cornerstone for defining the clinical problem, guiding differential diagnoses, and formulating appropriate treatment plans [28,29]. A meticulously conducted history can yield more diagnostic insights than a physical examination, fostering a trusting relationship between the patient and healthcare provider [30]. Conversely, the physical examination complements history-taking by enabling clinicians to evaluate the patient's physical signs, localize lesions, and refine diagnostic hypotheses [29]. In disciplines like neurology, the examination is pivotal in localizing lesions within the central or peripheral nervous system, facilitating etiological diagnosis and treatment planning [29]. The neurological examination, in particular, represents a distinct exercise in clinical medicine, necessitating specific techniques to detect conditions that may not be visibly apparent or palpable [29].

Imaging Modalities: Chest X-ray, Echocardiography, MRI, CT

Chest X-ray is a primary imaging modality for initial evaluation, commonly employed to reveal evidence of underlying cardiopulmonary diseases, pulmonary hypertension, or right ventricular (RV) enlargement [31]. It offers a comprehensive overview of the chest area, facilitating the identification of abnormalities suggestive of cor pulmonale. Echocardiography holds essential significance in assessing disease and RV function, aiding in delineating the etiology of pulmonary hypertension and cor pulmonale [31]. By providing valuable insights into the heart's structure and function, echocardiography contributes significantly to diagnosing and managing cor pulmonale. MRI (Magnetic Resonance Imaging) emerges as a noninvasive imaging modality devoid of ionizing radiation, crucial for evaluating RV structure, remodeling, and function in cor pulmonale patients [32]. Its utility lies in assessing pulmonary artery dimensions, offering advantages over traditional echocardiography. CT (Computed Tomography) scans play a pivotal role in detecting lung pathologies and alternative causes of pulmonary hypertension, encompassing lung parenchymal disorders, thromboembolic disease, and vascular abnormalities like pulmonary vein stenosis [33]. CT angiography distinguishes various cor pulmonale conditions, facilitating accurate diagnosis and management.

Cardiopulmonary Exercise Testing

Cardiopulmonary exercise testing (CPET) is a valuable diagnostic modality to assess a patient's functional capacity by evaluating the integrative exercise responses involving the pulmonary, cardiovascular, and skeletal muscle systems [34-36]. This non-invasive test provides a dynamic evaluation of exercise performance, functional capacity, and impairment, offering insights into undiagnosed exercise intolerance and cardiovascular and pulmonary diseases [36]. CPET entails measuring respiratory oxygen uptake (VO2), carbon dioxide production (VCO2), and ventilatory measures during exercise to assess both submaximal and peak exercise responses [34,36]. Increasingly, CPET finds application across a broad spectrum of clinical scenarios, aiding in assessing exercise tolerance and prognostication in heart failure patients and informing treatment decisions based on functional capacity and impairment [36]. The rationale for conducting CPET encompasses detecting suspected cardiopulmonary diseases, evaluating disease severity, determining treatment needs such as supplemental oxygen, identifying exercise-induced asthma, and monitoring symptoms like chest pain, dizziness, and dyspnea [34]. CPET plays a crucial role in determining patients' functional capacity and impairment, facilitating the evaluation of progressive diseases, treatment efficacy, and rehabilitation programs [35,37]. During the test, patients engage in physical exercise on either a treadmill or bicycle ergometer, with various exercise protocols tailored to individual patients based on their health status and level of fitness [36].

Laboratory Investigations: Arterial Blood Gases, Pulmonary Function Tests

Laboratory investigations of arterial blood gases (ABG) and pulmonary function tests (PFTs) play pivotal roles in assessing respiratory function and diagnosing lung conditions. ABG testing evaluates oxygen, carbon dioxide levels, and acid-base balance in the blood, furnishing crucial information for diagnosing lung and breathing disorders, acid-base imbalances, and kidney problems [38,39]. Conducted using blood drawn from an artery, typically the radial artery in the wrist, ABG tests are indispensable for accurately monitoring and diagnosing respiratory conditions [39]. Conversely, pulmonary function tests (PFTs) gauge lung function by assessing oxygen transfer efficiency into the blood and carbon dioxide removal, aiding in diagnosing conditions such as asthma, COPD, lung cancer, and other respiratory ailments [40]. PFTs encompass tests like spirometry, which measures air volume and flow rates, and body plethysmography, which evaluates lung volume and airway resistance [41]. These noninvasive tests offer valuable insights into lung health and function, serving as vital tools for diagnosing and monitoring various respiratory conditions.

Hemodynamic Assessment: Right Heart Catheterization

Hemodynamic assessment via right heart catheterization represents a critical procedure for evaluating patients' hemodynamic status, particularly in conditions such as cardiogenic shock and pulmonary arterial hypertension (PAH). This procedure facilitates a comprehensive evaluation of the hemodynamic state, furnishing vital information for diagnosis and prognosis and guiding treatment decisions [42,43]. Right heart catheterization entails the insertion of a catheter into the right side of the heart to measure pressures and obtain hemodynamic parameters, including cardiac output, pulmonary artery pressures, and pulmonary capillary wedge pressure [42,43]. It proves instrumental in assessing conditions such as pulmonary hypertension, shock, fluid management, pericardial tamponade, and distinguishing between cardiomyopathies [44]. The procedure adheres to a systematic approach in collecting and interpreting hemodynamic data, ensuring accurate assessment and informed management decisions [44]. Standardizing the acquisition and interpretation of right heart catheterization results holds paramount importance for optimal patient care, particularly in scenarios involving advanced heart failure and cardiogenic shock [42]. By establishing protocols and focusing on physiological processes and thresholds, healthcare providers can enhance their understanding and interpretation of hemodynamic assessments, thereby fostering improved outcomes and survival rates [42].

Management strategies

Pharmacological Interventions

Pharmacological interventions for pediatric cor pulmonale primarily involve vasodilators aimed at managing pulmonary hypertension and enhancing right ventricular function. Treatment options encompass targeted pulmonary vasodilator therapies, including endothelin receptor antagonists, prostacyclin analogs, and phosphodiesterase type 5 inhibitors [45]. These medications have demonstrated hemodynamic and functional improvements in children with pulmonary arterial hypertension (PAH) [45]. Specific drugs utilized in managing pediatric cor pulmonale comprise oral calcium channel blockers like nifedipine, continuous intravenous prostacyclin, bosentan, sildenafil (Revatio), and sitaxsentan [45,46]. Sildenafil is indicated for pediatric patients aged 1 to 17 years to ameliorate exercise ability and pulmonary hemodynamics in PAH [46]. FDA-approved for idiopathic or congenital PAH in children aged 3 years and older, bosentan aims to enhance pulmonary vascular resistance and exercise capacity [46]. Additionally, theophylline, in addition to its bronchodilatory effects, has been reported to acutely reduce pulmonary vascular resistance and pulmonary arterial pressures in patients with chronic cor pulmonale secondary to COPD [31]. It also exhibits a weak inotropic effect and may enhance right and left ventricular ejection [31]. Pharmacological interventions for pediatric cor pulmonale concentrate on vasodilators to tackle pulmonary hypertension and enhance right ventricular function, with medications such as endothelin receptor antagonists, prostacyclin analogs, phosphodiesterase type 5 inhibitors, and specific drugs like sildenafil and bosentan playing pivotal roles in managing this condition.

Diuretics play a significant role in chronic cor pulmonale management, particularly when right ventricular (RV) filling volume is markedly elevated and in addressing associated peripheral edema [31]. These agents may improve the function of both the right and left ventricles [31]. However, caution must be exercised in diuretic use, as excessive volume depletion may precipitate a decline in cardiac output [31]. Another potential complication of diuretic therapy is the induction of hypokalemic metabolic alkalosis, which diminishes the effectiveness of carbon dioxide stimulation on respiratory centers and reduces ventilatory drive [31]. Adverse electrolyte and acid-base effects of diuretics can also precipitate cardiac arrhythmias, thereby compromising cardiac output. While diuretics are recommended in chronic cor pulmonale management, they necessitate cautious administration to avert potential complications such as volume depletion, electrolyte imbalances, and cardiac arrhythmias [47].

Pharmacological interventions for heart conditions frequently incorporate inotropic agents, which are medications impacting the contraction of the heart muscle. These agents can be divided into positive inotropes, bolstering the force of heart muscle contraction, and negative inotropes, diminishing the force of muscular contractions, thereby reducing the heart's workload [48]. Positive inotropes find application in conditions like congestive heart failure, cardiomyopathy, and post-heart attack scenarios to enhance the heart's pumping capacity with fewer beats [49]. Conversely, negative inotropes are employed in conditions such as hypertension, chronic heart failure, abnormal heart rhythms, and angina to alleviate stress on the heart and avert future heart attacks [49]. The utilization of inotropic agents in heart failure treatment, particularly acute decompensated systolic heart failure, is firmly established [50]. These agents enhance myocardial contractility and can be pivotal in maintaining hemodynamic stability in patients with low cardiac output and end-organ dysfunction [50]. Commonly used inotropic drugs include digoxin, dopamine, dobutamine, norepinephrine, milrinone, levosimendan, and omecamtiv mecarbil, with considerations given to their mechanisms of action, indications, and potential risks [50]. In clinical practice, inotropic agents are administered for acute heart failure concurrent with hypoperfusion due to decreased cardiac output, aiming to stabilize hemodynamics and restore peripheral perfusion [51]. Initially prescribed for short durations, their use may be extended as a bridge to definitive treatments like heart transplants or mechanical circulatory support [51]. Physicians exercise caution due to potential adverse events associated with inotropic agents, tailoring their use based on patient-specific indications and dosing strategies [51].

Pharmacological interventions for cor pulmonale, particularly anticoagulants, are pivotal in managing the condition. In the context of cor pulmonale, anticoagulation therapy is indicated in cases of thromboembolic events to forestall further complications and enhance outcomes [52]. Anticoagulants mitigate the risk of blood clots, which can exacerbate cor pulmonale progression and give rise to complications like pulmonary embolism [31]. Incorporating anticoagulants is integral to a comprehensive treatment approach aimed at ameliorating oxygenation, augmenting right ventricular function, and mitigating pulmonary vasoconstriction in cor pulmonale patients [52]. While the advantageous effects of anticoagulants in cor pulmonale management are evident, their use necessitates vigilant monitoring and customization to each patient's specific needs and risk factors [52].

Non-pharmacological Interventions

Oxygen therapy is a fundamental non-pharmacological intervention in pediatric cor pulmonale management. Particularly crucial for enhancing oxygenation levels, it proves invaluable for hypoxic patients, alleviating hypoxemia, lessening the heart's workload, and enhancing overall oxygen delivery to tissues [53]. Continuous long-term oxygen therapy is recommended for cases of chronic cor pulmonale with severe polycythemia resulting from chronic hypoxia [54]. In acute exacerbations of cor pulmonale, oxygen therapy assumes a pivotal role alongside hospitalization, heart failure control, cardiac arrhythmia management, infection control, and tailored treatment for underlying lung disease. It constitutes a cornerstone intervention significantly impacting prognosis and enhancing the quality of life for pediatric patients with cor pulmonale [55].

Mechanical ventilation is another critical non-pharmacological intervention in managing respiratory failure associated with pediatric cor pulmonale. Especially pertinent when non-invasive ventilation (NIV) proves inadequate, mechanical ventilation is instrumental in minimizing ventilator-associated lung injury by meticulously managing parameters to mitigate barotrauma and polytrauma [56]. NIV is preferred initially with a nasal or facial mask, enhances gas exchange, reduces respiratory rate, and alleviates breathlessness severity [56]. NIV also contributes to lowered complication rates like ventilator-associated pneumonia, potentially leading to reduced mortality rates among affected children. However, in instances of NIV failure, invasive ventilation becomes indispensable as a rescue therapy, furnishing adequate respiratory support and enhancing outcomes [56].

Pulmonary rehabilitation is a vital non-pharmacological intervention tailored to address cor pulmonale management. Aimed at enhancing exercise capacity, quality of life, and symptoms in patients with pulmonary arterial hypertension (PAH), these programs typically integrate exercise training, education, and psychosocial support aligned with individual patient needs [57]. Exercise training, in particular, holds promise as a non-pharmacological therapy, with home-based rehabilitation programs proving effective in bolstering function and quality of life [57]. Focused on structured, safe exercise regimens tailored to patient conditions, these programs enhance physical capacity and overall well-being.

Surgical interventions such as pulmonary artery banding and lung transplantation are significant non-pharmacological measures in pediatric cor pulmonale management. Pulmonary artery banding finds indication in chronic cor pulmonale cases with severe polycythemia triggered by chronic hypoxia [58]. Lung transplantation consideration arises in select cases, particularly when prior interventions yield limited success or, in severe instances, necessitate intervention due to refractory pulmonary disease [59]. These surgical interventions are crucial in managing pediatric cor pulmonale by addressing underlying pathology and ameliorating the overall prognosis for affected children. Pulmonary artery banding mitigates strain on the right ventricle induced by chronic hypoxia, while lung transplantation offers a definitive solution in cases of severe, treatment-resistant pulmonary disease [60].

Long-Term Management and Follow-Up Care

Long-term management and follow-up care for pediatric cor pulmonale necessitate a comprehensive approach addressing the underlying pulmonary disease, optimizing oxygenation, and managing right ventricular dysfunction. In chronic scenarios, medical therapy prioritizes treating the primary pulmonary disease, improving oxygen levels, enhancing right ventricular function, and reducing pulmonary vasoconstriction [55]. This entails employing modalities such as oxygen therapy, diuretics, vasodilators, digitalis, theophylline, and anticoagulation therapy to manage chronic cor pulmonale [55] effectively. Surgical interventions may be warranted in specific cases, like phlebotomy for chronic cor pulmonale accompanied by severe polycythemia, and lung transplantation for advanced stages of diseases complicated by cor pulmonale such as idiopathic pulmonary fibrosis or cystic fibrosis [61]. Phlebotomy can lower mean pulmonary artery pressure and vascular resistance, enhancing exercise performance in patients with chronic cor pulmonale and chronic hypoxia-induced severe polycythemia [62]. In outpatient settings, diligent monitoring is indispensable, encompassing regular assessment of oxygen requirements, pulmonary function, and consideration of pulmonary rehabilitation programs to optimize patient outcomes. Patient education emphasizing adherence to medical therapy is pivotal for improving mortality and morbidity by effectively managing hypoxia and underlying medical conditions [63]. For pediatric patients with bronchopulmonary dysplasia (BPD), a frequent cause of cor pulmonale, a structured follow-up protocol is recommended to monitor lung function, growth, and neurodevelopmental outcomes throughout adolescence and adulthood. This protocol includes pre-discharge assessments, oxygen therapy guidelines, imaging tests like chest CT for lesion detection, and screening for pulmonary hypertension using echocardiography. Regular follow-up visits, diagnostic tests, and adherence to treatment recommendations constitute vital components of long-term management and follow-up care for pediatric cor pulmonale [64].

Future directions and research opportunities

Advances in Understanding the Pathophysiology of Pediatric Cor Pulmonale

Significant strides have been made in understanding the pathophysiology of pediatric cor pulmonale, revealing crucial factors contributing to this condition. Chronic hypoxia emerges as a central player, initiating a cascade of events, including endothelial dysfunction, pulmonary vasoconstriction, heightened pulmonary artery pressure, and increased pulmonary vascular resistance [65]. Ultimately, these mechanisms culminate in right ventricular failure secondary to pulmonary complications, distinctly different from right heart failure linked with congenital heart defects or primary pulmonary vascular disease [65]. Notably, in pediatric patients with obstructive sleep apnea (OSA) and cor pulmonale, distinct clinical and hemodynamic traits have surfaced, underscoring OSA's impact on cor pulmonale development [21]. Studies reveal that children with OSA and cor pulmonale exhibit elevated arousal indexes, decreased oxygen saturation levels, and a heightened incidence of bradycardia events compared to counterparts without cor pulmonale [21]. Adenotonsillectomy emerges as a significant intervention, substantially reducing pulmonary arterial pressure in children with OSA and cor pulmonale, highlighting the criticality of early management [21]. Furthermore, insights into the pathophysiology of childhood interstitial lung disease (chILD) shed light on structural lung tissue remodeling, compromised gas exchange, and pulmonary hypertension development, all contributing to cor pulmonale progression in pediatric populations [66]. Sustained hypoxemia in chILD can instigate pulmonary hypertension and vascular remodeling, fostering cor pulmonale advancement in affected children [66]. The diverse spectrum of chILD entities underscores the intricacies of pediatric lung ailments, advocating for tailored diagnostic and management strategies grounded in specific underlying pathophysiologic mechanisms [66].

Emerging Therapies and Treatment Modalities

Recent advancements in therapies and treatment approaches for pediatric cor pulmonale primarily focus on enhancing outcomes for children diagnosed with pulmonary arterial hypertension (PAH). While several drugs have been approved for adult patients, their efficacy and safety in pediatric populations remain inadequately studied [67]. However, the landscape of PAH management in children has undergone significant transformations with new medications and the establishment of specialized services such as the United Kingdom Pulmonary Hypertension Service for Children, which has streamlined diagnosis and care provision [68]. A notable shift in therapeutic strategies involves the development of medications aimed at restructuring the pulmonary vasculature toward a more normalized state rather than solely dilating the diseased pulmonary vascular bed [68]. This includes novel endothelin receptor antagonists, phosphodiesterase inhibitors, and prostacyclin analogs, which have demonstrated efficacy when administered orally [68]. Furthermore, emerging prostanoids like ralinepag, an oral IP (prostaglandin I2) receptor agonist, have exhibited promising results in reducing pulmonary vascular resistance compared to placebo in PAH patients [69]. Anticipated future investigations are poised to explore alternative agents such as inhaled nitric oxide, additional phosphodiesterase inhibitors, substrate loading agents, and elastase inhibitors, aiming to expand further treatment options for children afflicted with PAH [70]. As knowledge accumulates and experience grows with these innovative therapeutic agents, the therapeutic algorithm for managing PAH in children is expected to undergo further refinement and evolution [70].

Areas Requiring Further Investigation and Clinical Trials

Further investigation and clinical trials in pediatric pulmonary hypertension are imperative to address crucial gaps in knowledge and improve patient care. One key area of focus is the necessity for more randomized double-blind trials to generate essential data on the safety, efficacy, and optimal dosing of therapies for children with pulmonary arterial hypertension (PAH) [71]. Given the rarity of pediatric pulmonary hypertension cases and developmental differences across childhood, larger, multicenter trials are essential to ensure comprehensive evaluation and generalizability of findings [72]. Research opportunities also extend to defining the natural history, epidemiology, and course of pediatric pulmonary vascular disease (PVD) across diverse conditions [72]. Novel imaging or physiologic approaches are needed to assess lung vascular and alveolar growth to enhance diagnostic accuracy and therapeutic monitoring in pediatric patients [72]. Additionally, efforts should be directed toward characterizing unique aspects of the developing lung circulation, identifying barriers to clinical trials in children with pulmonary hypertension, and establishing biomarkers to predict disease risk, severity, and progression [71]. Understanding the natural history, fundamental mechanisms, and treatment of childhood pulmonary hypertension is paramount, given the significant differences from adult PH in vascular function, genetics, and treatment response [72]. Defining the natural history, course, and optimal management strategies for pediatric cor pulmonale is vital to improve outcomes and enhance the quality of care for affected children. By addressing these research priorities, clinicians and researchers can advance the understanding and treatment of pediatric pulmonary hypertension, ultimately improving the lives of affected children.

## Conclusions

In conclusion, pediatric cor pulmonale, characterized by right ventricular dysfunction due to chronic pulmonary hypertension, presents a significant challenge requiring early and precise diagnosis to prevent severe outcomes. The multifactorial etiology necessitates a comprehensive diagnostic approach, including advanced imaging, hemodynamic assessments, and thorough laboratory investigations. Effective management involves a combination of pharmacological and non-pharmacological interventions tailored to the underlying causes and disease severity, with long-term follow-up essential for monitoring and adjusting treatment. The complexity of pediatric cor pulmonale underscores the importance of multidisciplinary care, with collaboration among pediatric cardiologists, pulmonologists, critical care specialists, and other healthcare professionals crucial for optimal patient outcomes. Continuous advancements in diagnostics, therapeutics, and our understanding of the disease's pathophysiology are vital. Future research should focus on innovative treatment approaches and clinical trials to refine evidence-based guidelines, aiming to improve the quality of care and outcomes for children affected by this condition.
